# 8-Oxo-9-Dihydromakomakine Isolated from *Aristotelia chilensis* Induces Vasodilation in Rat Aorta: Role of the Extracellular Calcium Influx

**DOI:** 10.3390/molecules23113050

**Published:** 2018-11-21

**Authors:** Fredi Cifuentes, Javier Palacios, Adrián Paredes, Chukwuemeka R. Nwokocha, Cristian Paz

**Affiliations:** 1 Laboratorio de Fisiología Experimental, Instituto Antofagasta, Universidad de Antofagasta, Antofagasta 1270300, Chile; fredi.cifuentes@uantof.cl; 2 Facultad Ciencias de la Salud, Instituto de EtnoFarmacología (IDE), Universidad Arturo Prat, Iquique 1110939, Chile; 3 Laboratorio de Química Biológica, Instituto Antofagasta, Universidad de Antofagasta, Antofagasta 1270300, Chile; adrian.paredes@uantof.cl; 4 Departamento de Química, Facultad de Ciencias Básicas, Universidad de Antofagasta, Antofagasta 1270300, Chile; 5 Department of Basic Medical Sciences Physiology Section, Faculty of Medical Sciences, The University of the West Indies, Mona, Kingston 7, KGN, Jamaica (W.I.); chukwuemeka.nwokocha@uwimona.edu.jm; 6 Departamento de Ciencias Químicas y Recursos Naturales, Facultad de Ingeniería, Universidad de La Frontera, Temuco 4780000, Chile

**Keywords:** *Aristotelia chilensis* Molina Stuntz, vascular activity, endothelium-independent, indole alkaloid, 8-oxo-9-dihydromakomakine, voltage-dependent calcium channels

## Abstract

8-Oxo-9-dihydromakomakine is a tetracyclic indole alkaloid extracted from leaves of the Chilean tree *Aristotelia chilensis*. The present study investigated the effects of this alkaloid on vascular response in tissues isolated from aortic segments obtained from normotensive rats. Our results showed that 8-oxo-9-dihydromakomakine induced a dose-dependent relaxation of aortic rings pre-contracted with phenylephrine (PE; 10^−6^ M). The vasorelaxation induced by 8-oxo-9-dihydromakomakine in rat aortic rings is independent of endothelium. The pre-incubation of aortic rings with 8-oxo-9-dehydromakomakine (10^−4^ M) significantly reduced the contractile response to KCl (*p* < 0.001) more than PE (*p* < 0.05). The highest dose of 8-oxo-9-dehydromakomakine (10^−4^ M) drastically reduced the contraction to KCl (6·10^−2^ M), but after that, PE (10^−6^ M) caused contraction (*p* < 0.05) in the same aortic rings. The addition of 8-oxo-9-dihydromakomakine (10^−5^ M) decreased the contractile response to tetraethylammonium (a voltage-dependent potassium channels blocker; TEA; 5 × 10^−3^ M; *p* < 0.01) and BaCl_2_ (a non-selective inward rectifier potassium channel blocker; 5 × 10^−3^ M; *p* < 0.001) in rat aorta. 8-oxo-9-dihydromakomakine (10^−5^ M) decreased the contractile response to PE in rat aorta in the presence or absence of ouabain (an inhibitor of Na,K-ATPase; 10^−3^ M; *p* < 0.05). These results could indicate that 8-oxo-9-dihydromakomakine partially reduces plasma membrane depolarization-induced contraction. In aortic rings depolarized by PE, 8-oxo-9-dihydromakomakine inhibited the contraction induced by the influx of extracellular Ca^2+^ in a Ca^2+^ free solution (*p* < 0.01). 8-oxo-9-dihydromakomakine reduced the contractile response to agonists of voltage-dependent calcium channels type L (Bay K6844; 10^−8^ M; *p* < 0.01), likely decreasing the influx of extracellular Ca^2+^ through the voltage-dependent calcium channels. This study provides the first qualitative analysis indicating that traditional folk medicine *Aristotelia chilensis* may be protective in the treatment of cardiovascular pathologies.

## 1. Introduction

Maqui (*Aristotelia chilensis* (Molina) Stuntz, *Elaeocarpaceae*) is a Chilean native tree sacred to the Araucanian people. Nowadays, maqui fruits have aroused special interest due to their beneficial properties for human health, such as antioxidant, cardioprotective, anti-inflammatory, and enzymatic activities [1,2,3]. These bioactivities could be useful for the treatment of vascular diseases such as hypertension, myocardial ischemia, and cerebral infarction, which have become worldwide epidemics in modern society [4].

*Aristotelia chilensis* produce various active components including a high concentration of flavonoids in fruits [1,2]. Chemical analysis have reported that maqui leaves consists of non-iridoid monoterpene indole alkaloids and polyphenolic compounds [5,6]. It is known that several pure compounds from plants may modulate the vascular response, and therefore act as antihypertensives. In fact, some flavonoids (quercetin or myricetin) cause vasoconstriction because they activate Ca_v_1.2 channel, while flavonols (kaempferol and galangin), a flavone (chrysin), and an isoflavone (genistein) inhibit Ca_v_3.1 channel by producing vasorelaxation [7]. In addition, galangin and chrysin can inhibit Ca_v_1.2 channel [8].

However, there are a paucity of data and studies about the vascular response of alkaloids from *A. chilensis*. In order to have a more comprehensive idea of the pharmacological activity of alkaloids produced by *A. chilensis* on the cardiovascular system, the main component of leaves of maqui was purified and chemically characterized by NMR spectroscopy as 8-oxo-9-dihydromakomakine. Then, its activity was determined by the induction of tension change in vascular tissue isolated from aortic segments obtained from normotensive rats. The potential mechanisms were evaluated from the endothelium and nitric oxide (NO) production, together with the role of cytosolic calcium, and its modulation by calcium channels, potassium channels, and Na,K-ATPase.

## 2. Results

### 2.1. 8-Oxo-9-Dihydromakomakine Induced Vasodilation in Rat Aorta

**Figure 1:**
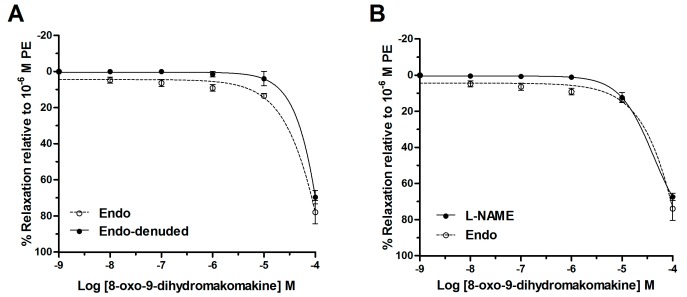
Vasorelaxation effect of 8-oxo-9-dihydromakomakine in intact (Endo) and denuded-endothelium (Endo-denuded) aortic rings (**A**), and in presence of 10^−4^ M l-NAME in the intact aorta (**B**). Values are mean ± standard error of the mean of 6 experiments. Two-way ANOVA followed by Bonferroni’s post-hoc test.

8-Oxo-9-dehydromakomakine induced vascular relaxation on aortic rings pre-contracted with PE (10^−6^ M). In fact, 8-oxo-9-dehydromakomakine caused relaxation in intact aorta and endothelium-denuded aorta (Figure 1A). This result was confirmed because 8-oxo-9-dehydromakomakine also produced relaxation in aortic rings pre-incubated with 10^−4^ M *N*^ω^-nitro-l-arginine methyl ester (l-NAME) (Figure 1B).

### 2.2. 8-Oxo-9-Dihydromakomakine Reduced the Contractile Response to KCl and PE

**Figure 2:**
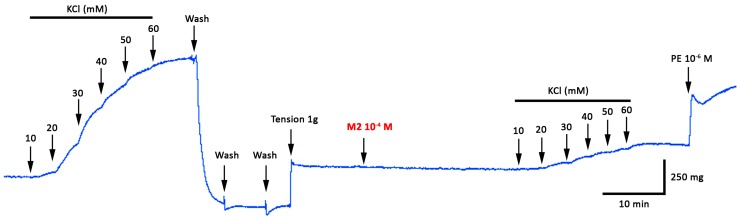
Original trace showing the time course of the concentration–response curves to KCl in intact aortic rings from male rats pre-incubated with M2 (8-oxo-9-dihydromakomakine; 10^−4^ M) for 20 min. Phenilephrine (PE; 10^−6^ M) increased drastically the contraction after the contractile response to 60 mM KCl.

The effect of 8-oxo-9-dihydromakomakine on contractile response induced by KCl and PE was studied in a new experiment (Figure 2).

**Figure 3:**
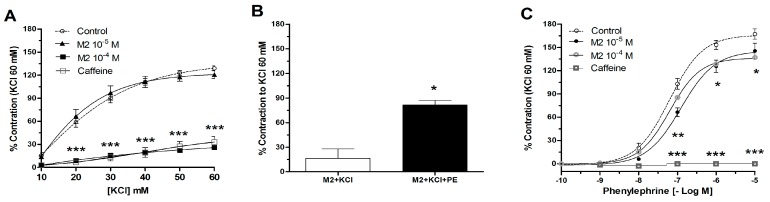
8-Oxo-9-dihydromakomakine (M2) decreased the contraction induced with KCl and PE in aortic rings of rat. Contractile responses to KCl (**A**) and PE (**C**) are expressed in relation to the maximal response of 60 mM of KCl. 10^−6^ M PE induced a contraction after 60 mM KCl-induced contraction in aortic rings pre-incubated with 10^−4^ M 8-oxo-9-dehydromakomakine (**B**). The aortic rings were pre-incubated with 10^−4^ M 8-oxo-9-dehydromakomakine or 2 × 10^−2^ M caffeine for 20 min. Values are mean ± standard error of the mean of 5–10 experiments. Significant differences (SEM) are represented with * *p* < 0.05, ** *p* < 0.01, *** *p* < 0.001 versus the control. Two-way ANOVA followed by Bonferroni’s post-hoc or test Student’s *t*-test.

Aortic rings were pre-incubated in absence and the presence of 8-oxo-9-dihydromakomakine (10^−5^ M and 10^−4^ M), before the addition of cumulative concentration of KCl (10 to 60 mM) and PE (10^−10^ to 10^−5^ M) (Figure 3). Results showed that the pre-incubation of aortic rings with 8-oxo-9-dihydromakomakine reduced the contractile response to KCl and PE. The pre-incubation with 8-oxo-9-dihydromakomakine decreased the maximal contraction (E_max_) to 60 mM KCl (130 ± 3% control vs. 26 ± 1% with 10^−4^ M 8-oxo-9-dihydromakomakine; *p* < 0.001; Figure 3A). The maximal response to PE (10^−5^ M) decreased from 170 ± 7% for control to 137 ± 24% in rings pre-incubated with 8-oxo-9-dihydromakomakine (10^−4^ M, *p* < 0.05; Figure 3C).

Interesting, we observed that the addition of 10^−6^ M PE significantly increased (*p* < 0.05) a 52 ± 11% contractile response in aortic rings pre-contracted with 60 mM KCl in presence of 10^−4^ M 8-oxo-9-dehydromakomakine (Figure 2 and Figure 3B). Also, caffeine (a methylxanthine alkaloid) significantly decreased the maximal contraction (E_max_) to 60 mM KCl (33 ± 5 % with 2 × 10^−2^ M caffeine; *p* < 0.001; Figure 3A) and 10^−5^ M PE (1 ± 2 % with 2 × 10^−2^ M caffeine; *p* < 0.001; Figure 3C).

### 2.3. Role of Potassium Channels in the Vascular Response to 8-Oxo-9-Dihydromakomakine

**Figure 4:**
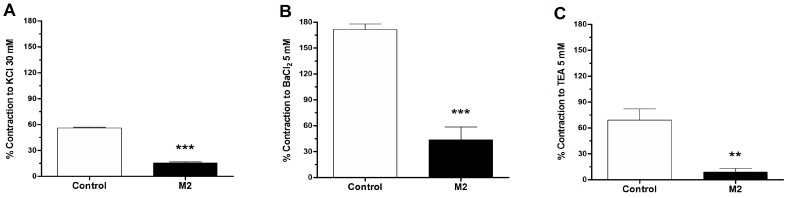
Effect of 8-oxo-9-dihydromakomakine on potassium channels. The pre-incubation of aortic rings with 10^−5^ M 8-oxo-9-dihydromakomakine significantly decreased the contractile response to 30 mM KCl (**A**), 5 × 10^−3^ M BaCl_2_ (**B**), and 5 × 10^−3^ M TEA (**C**). Values are mean ± standard error of the mean of 6 experiments. Significant differences (SEM) are represented with ** *p* < 0.01, *** *p* < 0.001 versus the control. Student’s *t*-test.

To study the role of potassium channels on the depolarization-induced contraction to KCl, BaCl_2_, and TEA, aortic rings of rat were pre-incubated with 8-oxo-9-dihydromakomakine. The contraction was induced by 30 mM KCl, 5 × 10^−3^ M BaCl_2_, and 5 × 10^−3^ M TEA for 10 min. The aortic rings were pre-incubated with 8-oxo-9-dihydromakomakine (10^−5^ M) for 20 min, and showed a significantly lower contractile response to KCl (*p* < 0.001; Figure 4A), BaCl_2_ (*p* < 0.001; Figure 4B), and TEA (*p* < 0.01; Figure 4C) than the control.

### 2.4. Role of Na,K-ATPase in the Vascular Response to 8-Oxo-9-Dihydromakomakine

**Figure 5:**
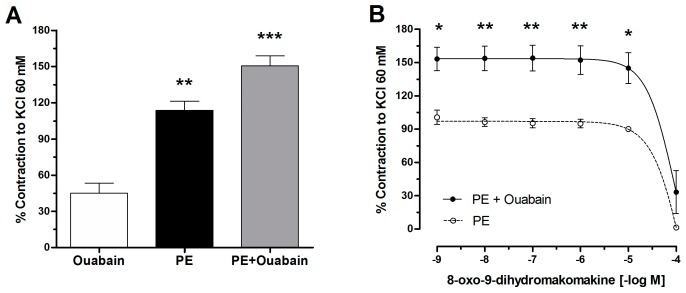
Effect of 8-oxo-9-dihydromakomakine on Na,K-ATPase function. Contractile response to Ouabain, PE and PE+Ouabain before adding 8-oxo-9-dihydromakomakine (**A**). The aortic rings were pre-incubated with 10^−3^ M ouabain for 20 min, and 8-oxo-9-dihydromakomakine was added in cumulative doses on aortic rings pre-contracted with 10^−6^ M PE (**B**). Values are mean ± standard error of the mean of 4 experiments. Significant differences (SEM) are represented with * *p* < 0.05, ** *p* < 0.01, *** *p* < 0.001 versus Ouabain or PE.

To evaluate the role of the plasma membrane depolarization caused by the inhibition of Na,K-ATPase on the contractile response to PE, aortic rings were pre-incubated with ouabain (Figure 5). Cumulative doses of 8-oxo-9-dihydromakomakine (10^−9^ M to 10^−4^ M) were added to intact aortic rings pre-contracted with 10^−6^ M PE in the presence or absence of ouabain 10^−3^ M (Figure 5B). The pre-incubation of aortic rings with 10^−3^ M ouabain (an inhibitor of Na,K-ATPase) did not reduce the relaxation of 10^−4^ M 8-oxo-9-dihydromakomakine in aortic rings pre-contracted with 10^−6^ M PE.

### 2.5. Role of Extracellular Calcium in the Vascular Response to 8-Oxo-9-Dihydromakomakine

**Figure 6:**
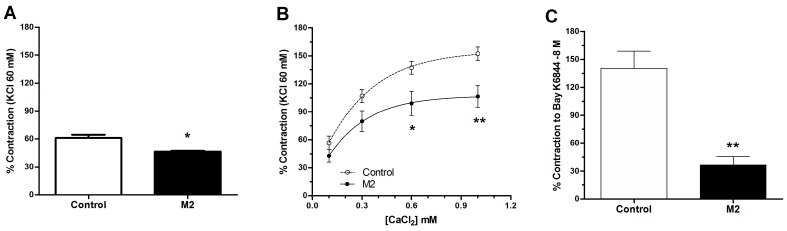
Effect of 8-oxo-9-dihydromakomakine (M2) on the calcium current blockage in calcium-free medium. The aortic rings were pre-incubated in a free calcium buffer for 10 min before PE was added (**A**), and then, the CaCl_2_ (0.1, 0.3, 0.6, and 1.0 × 10^−3^ M) was added to the bath (**B**). Vasoconstriction occurred just when the agonist calcium channel (10^−8^ M Bay K6844) was added with 15 × 10^−3^ M KCl to the bath (**C**). Values are mean ± standard error of the mean of 6 experiments. Significant differences (SEM) are represented with * *p* < 0.05, ** *p* < 0.01. Student’s *t*-test or two-way ANOVA followed by Bonferroni’s post-hoc test.

We studied calcium fluxes to determine if the contractions induced by PE in the presence of 8-oxo-9-dihydromakomakine could be modulated by calcium inflow from the extracellular space. Thus, we added increasing concentrations of CaCl_2_ to a calcium-free medium (Figure 6).

Figure 6 shows that aortic rings pre-incubated with 10^−5^ M of 8-oxo-9-dihydromakomakine decreased contractile response to PE (10^−6^ M) in free calcium medium with values of 61 ± 3% control vs. 46 ± 1% (*p* < 0.05; Figure 6A) and presence of CaCl_2_ in cumulative dose (0.1 to 1.0 × 10^−3^ M) with maximum values of 152 ± 21% control vs. 107 ± 14% with 1.0 × 10^−3^ M CaCl_2_ (** *p* < 0.01; Figure 6B). To study the participation of calcium channels, an agonist of voltage-dependent calcium channels type L (Bay K6844) was used. As shown in Figure 6C, we confirmed that 10^−5^ M 8-oxo-9-dihydromakomakine significantly reduced the contractile response to 10^−8^ M Bay K6844 (140 ± 19% control vs. 37 ± 9% with 10^−5^ M M2; *p* < 0.01). 

### 2.6. Chemical Characterization of 8-Oxo-9-Dihydromakomakine

The compound was formed by four fused rings, with a planar region composed by an indole moiety, and linked to two aliphatic rings by a ketone. The ^1^H-NMR (Figure S1) and ^13^C-NMR (Figure S2) results are in excellent agreement with previous data obtained by X-ray studies [6].

8-oxo-9-dihydromakomakine formula; C_20_H_22_N_2_O, yellow crystals, mp 259–260 °C, [α]_D_^25^ + 16.8 (CHCl_3_, c 0.24), IR (cm^−1^): 3248, 2970, 2938, 2431, 1589, 1504: ESIMS (M + 1) 307.1654.

## 3. Discussion

*Aristotelia chilensis* or Maqui is used in Chilean traditional medicine and nowadays its fruits are considered as “superfruit”, due to the high concentration of polyphenols that display antioxidant and cardioprotective bioactivities bringing benefits to human health [1,2]. Earlier studies from Mexican plants have shown that triterpenes and polyphenolic compounds have vasodilator effects [7], which could explain or suggest vasodilatory activity by the fruits of *A. chilensis*. However, there is no information about the cardiovascular activity of the metabolites isolated from the leaves of this plant. In the present study, we evaluated the vasodilatory activity of 8-oxo-9-dihydromakomakine, a natural tetracyclic indole alkaloid isolated from the leaves of *A. chilensis*, in search of natural compounds that could be used to develop new therapeutic agents to treat cardiovascular diseases.

Results showed than 8-oxo-9-dihydromakomakine induced relaxation in aortic rings pre-contracted with PE. The rings were obtained from normotensive rats and the relaxation was evidenced in a dose-dependent manner. The vasodilator activity of endothelium and the endothelial nitric oxide synthase (eNOS) were studied in two experiments. First, 8-oxo-9-dihydromakomakine caused vasodilation in intact and endothelium-denuded aortic rings when they were exposed to a cumulative concentrations of this one. Second, the inhition of the enzyme eNOS by pre-incubation of aortic rings with L-NAME did not alter the vasodilation caused by 8-oxo-9-dehydromakomakine. These results suggest that the vasodilation mechanism is independent of endothelium [8]. Thus, 8-oxo-9-dihydromakomakine goes through the endothelium and evoke a vascular response in the smooth muscle.

In agreement with above relaxation data, the pre-incubation of aortic rings with 8-oxo-9-dehydromakomakine significantly reduced the KCl-induced contractions more than PE-induced contractions. The pre-incubation with 8-oxo-9-dihydromakomakine significantly decreased the contractile response to KCl, BaCl_2_, and TEA in rat aorta. The KCl-induced contraction was caused by an increase of extracellular potassium, leading to membrane depolarization, which increases calcium influx from extracellular sources, involving voltage-dependent calcium channels [9]. BaCl_2_ and TEA block inward rectifying potassium channels [10], or potassium channels activated by calcium [11], respectively, thus depolarizing the plasma membrane and vasoconstriction. Similar results were obtained with the inhibition of Na,K-ATPase, which causes depolarization of plasma membrane and induced vasoconstriction [12]. 8-oxo-9-dihydromakomakine significantly decreased the contractile responses to PE in rat aorta in the presence or absence of ouabain. Therefore, these results could indicate that 8-oxo-9-dihydromakomakine partially reduces plasma membrane depolarization-induced contraction.

To assess the effect of 8-oxo-9-dihydromakomakine on the calcium current blockage, aortic rings of rat were pre-incubated with or without 8-oxo-9-dihydromakomakine, and kept on calcium-free medium. Contractions to PE were reduced in aortic rings pre-incubated with 8-oxo-9-dihydromakomakine in a free calcium medium, and by extracellular Ca^2+^ addition. Since PE stimulates inositol 1,4,5-trisphosphate receptor via protein kinase C [13] and releases calcium from intracellular store, which opens the store-operated calcium channels [14], our result analysis will suggest that the reduction of contractile response is mediated via a reduction of the influx of extracellular Ca^2+^ [15]. 

Recently, some studies showed that secondary compounds from medicinal plants can modulate voltage-dependent calcium channels, and thus, the vascular response. The pre-incubation with caffeine (a methylxanthine alkaloid) blunted contractile response to both, KCl and PE. The vasodilation mechanism of caffeine involves activation of the ryanodine channels [16], inhibition of the IP3 receptor [17], and voltage-dependent calcium channels [18,19]. Dicentrine (an aporphine alkaloid), dihidrocorynantheine, and tetrandrine (a bisbenzyl isoquinoline) cause inhibition of α_1_-adrenergic receptor and blocks calcium influx [20,21,22]. While, nantenine (an aporphine alkaloid) produces relaxation in aortic rings pre-contracted with noradrenaline or KCl [13]. We observed that 8-oxo-9-dihydromakomakine significantly reduced the contractile response to agonists of voltage-dependent calcium channels type L (Bay K6844), through a decrease of the calcium influx from extracellular sources [23]. In this way, it was confirmed that some alkaloid derivates may act as antihypertensives.

Since the aortic rings pre-incubated with the highest doses of 8-oxo-9-dehydromakomakine drastically reduced the contraction to KCl, and after responded to vasoactives substances, such as PE, it is possible to think that the high dose did not cause toxicity on vascular tissue [24]. Moreover, the effect induced by a toxic dose should be similar in vascular tissue pre-contracted with KCl or PE [25]; however, this was not observed in our study. Our use of aortic rings from rats to validate these findings are predicted on the observations that rat aorta assay provides a useful pharmacological tool for in vitro analysis, due to the low number of animals, good reproducibility of the experiments, and because the results are easily extrapolated to the in vivo models [26].

This study indicated that 8-oxo-9-dihydromakomakine reduces vascular tension endothelium-independently, and that the underlying mechanisms may involve decreases in the concentration of cytosolic Ca^2+^, likely through the blocking of voltage-dependent calcium channels.

## 4. Materials and Methods

### 4.1. Reagents and Chemicals

Silica gel GF254 analytical chromatoplates, Silica gel grade 60 A for column chromatography were purchased from Merck Co., Santiago, Chile. Precoated thin-layer chromatography TLC plates SIL G-100 UV254, 1.0 mm, preparative were purchased from Machery−Nagel (GmbH&Co, KG), Dueren, Germany. 8-oxo-9-dihydromakomakine was 99% purified through recrystallization.

The drugs used were l-phenylephrine hydrochloride (PE), *N*^ω^-nitro-l-arginine methyl ester (l-NAME), ouabain, and Bay K8644 (Sigma-Aldrich, St Luis, MO, USA). Caffeine, Tetraethylammonium (TEA), and Barium chloride dihydrate (BaCl_2_) were obtained from Merck (Darmstadt, Germany). The drugs were dissolved in distilled deionized water (deionized water Millipore) and kept at 4 °C. The stock solution of 8-oxo-9-dihydromakomakine was dissolved in DMSO (Merck, Germany). Final DMSO concentration in the organ bath was lower than 0.1%. The Krebs-Ringer bicarbonate (KRB) solutions were freshly prepared before each experiment.

### 4.2. Plant Material

Leaves of *A. chilensis* were collected on S 36°50′01.51″ W 73°01′53.75″, Concepción, Chile, during the end of the winter season. A voucher specimen was deposited at the herbal collection of the Laboratory of Natural Products (C. Paz), Universidad de La Frontera, Chile.

### 4.3. Apparatus

Optical rotations were recorded on a JASCO P-200 polarimeter (JASCO, Tokyo, Japan). Fourier-transform infrared (FTIR) spectra were measured on a Nicolet 6700 from Thermo Electron Corporation, (Madison, WI, USA) with the Attenuated Total Reflectance (ATR)-unit Smart Performer. Melting points were determined on a Melting Point SMP10 (Stuart, Staffordshire, UK) uncorrected. The ^1^H- and ^13^C-NMR spectra were recorded in CDCl_3_ solution in 5-mm tubes at room temperature (RT) on a Bruker Avance III spectrometer (Bruker Biospin GmbH, Rheinstetten, Germany) at 600.13 (^1^H) and 150.61 (^13^C) MHz, with the deuterium signal of the solvent as the lock and Tetramethylsilane (TMS; for ^1^H) or the solvent (for ^13^C) as internal standard. All spectra [^1^H, ^13^C, gradient-selected COrrelated SpectroscopY (gs-H,H-COSY), edited Heteronuclear Single Quantum Correlation (HSQC), and gradient-selected Heteronuclear Multiple Bond Correlation (gs-HMBC)] were acquired and processed with the standard Bruker software (Bruker, Rheinstetten, Germany). Tension changes in aortic rings were recorded with an Isometric force transducer (Radnoti, Monrovia, CA, USA), connected to a PowerLab 8/35 (ADInstruments, Bella Vista, Australia) for continuous recording of vascular tension using the LabChart Pro 8.1.2 computer program (ADInstruments, Bella Vista, Australia).

### 4.4. Isolation of 8-Oxo-9-Dihydromakomakine from Aristotelia chilensis

Fresh leaves of *A. chilensis* (7 kg) were crushed and extracted in acid water (pH 3, HCl) in the course of three days at room temperature. The aqueous layer was alkalinized to pH 11 with NaOH and subsequently extracted with EtOAc (3 × 1 L). The organic layer was evaporated at 45 °C and 200 mmHg to afford a gummy red residue. This total OH^−^ extract was chromatographed using a silica gel column (200−300 mesh) and increased solvent polarity (from hexane 100% to EtOAc 100%). The preparative chromatography was monitored by thin-layer chromatography (TLC; silica gel) and revealed using UV light, and later, Dragendorff’s reagent; those fractions showing similar TLC patterns were pooled and concentrated in vacuo. With the mixture of eluents hex/EtOAc (1:1) appeared a clear spot in TLC, then this fraction was purified by Sephadex LH-20 column (MeOH) and further recrystallization from EtOAc, giving 8-oxo-9-dihydromakomakine (85 mg, 0.0012% yield, yellow crystals).

### 4.5. Animals

For vascular reactivity experiments, Male Sprague-Dawley rats (6–8 weeks of age, 150–180 g) from the breeding colony at the Universidad de Antofagasta were used. All animals were housed in a temperature-controlled (21 ± 1 °C), light-cycled (08:00–20:00 h) room with *ad libitum* access to drinking water and standard rat chow (Champion, Santiago, Chile). In this study, 12 rats were randomly allocated. The investigation was conformed to the Guide for the Care and Use of Laboratory Animals published by the U.S. National Institute of Health (NIH Publication revised 2013), and the local animal research committee approved the experimental procedure used in the present study (number CEIC REV/2013).

### 4.6. Isolation of Aortic Rings

Rats were sacrificed through cervical dislocation. The thoracic aorta was quickly excised and placed in physiological KRB at room temperature containing (× 10^−3^ M): 4.2 KCl, 1.19 KH_2_PO_4_, 120 NaCl, 25 Na_2_HCO_3_, 1.2 MgSO_4_, 1.3 CaCl_2_, and 5 d-glucose (pH 7.4). Rings (3–5 mm and 2–4 mg) were prepared after connective tissue was cleaned out from the aorta, taking special care to avoid endothelial damage. Aortic rings were equilibrated for 40 min in KRB at 37 °C by constant bubbling with 95% O_2_ and 5% CO_2_.

### 4.7. Vascular Reactivity Experiments

Aortic rings from the same animal were studied in duplicate, using different vasoactive substances (PE, KCl). The rings were mounted on two 25-gauge stainless steel wires; the lower one was attached to a stationary glass rod and the upper one was attached to an isometric force transducer connected to a PowerLab 8/35. The vascular tension was recorded with LabChart Pro 8.1.2 computer program (ADInstruments, Bella Vista, Australia). After the equilibration period for 30 min, the aortic rings were stabilized by three successive near-maximum contractions with KCl (6 × 10^−2^ M) for 10 min. The passive tension on aorta was 1.0 g, which was determined to be the resting tension for obtaining maximum active tension induced by 6 × 10^−2^ M KCl [10].

### 4.8. Assessment of the Effects of 8-Oxo-9-Dihydromakomakine on the Vasodilation in Isolated Aortic Rings Pre-Contracted with PE, with and without Endothelium

Aortic rings were pre-contracted with 10^−6^ M PE, and then increasing concentrations 8-oxo-9-dihydromakomakine (10^−9^ to 10^−4^ M) were added to the bath. The endothelium removal was by gentle rubbing off the endothelium using a small piece of cotton. To evaluate the vascular function of the endothelium, the vasodilation to 10^−5^ M acetylcholine (ACh, muscarinic agonist) in pre-contracted aortic rings with 10^−6^ M PE was tested. According to the general use of rat aorta as a pharmacological tool for in vitro, the aortic rings were considered with a functional endothelial response if vasodilation was up to 70–80% [11].

After a steady contraction of the aortic rings with or without endothelium induced by PE (10^−6^ M) or KCl (6 × 10^−2^ M) was achieved, 8-oxo-9-dihydromakomakine (10^−9^ to 10^−4^ M), dissolved in DMSO (0.1% on the bath), was cumulatively added to the Krebs solution, and the results were used to generate a concentration‑response curve. In other protocols, an inhibitor of Na,K-ATPase (ouabain) was used. The aortic rings were pre-incubated with ouabain (10^−3^ M) for 20 min.

### 4.9. Assessment of the Effects of 8-Oxo-9-Dihydromakomakine on Endothelial Nitric Oxide Synthase (eNOS)

The role of endothelial nitric oxide (NO) in the rat aorta was studied. An inhibitor of eNOS (l-NAME) was used. The aortic rings were pre-incubated with L-NAME (10^−4^ M) for 20 min before the experiment. Then, the aortic rings were pre-contracted with 10^−6^ M PE, and then increasing concentrations 8-oxo-9-dihydromakomakine (10^−9^ to 10^−4^ M) were added to the bath.

### 4.10. Effect Calcium Dependence Extracellular Calcium Ionic and Effect of Barium chloride, Tetraethylammonium (TEA), and Bay K6844

To study the role of extracellular calcium, experiments were performed with a calcium-free KRB containing (× 10^−3^ M) 4.2 KCl, 1.19 KH_2_PO_4_, 125 NaCl, 25 NaHCO_3_, 1.2 MgSO_4_, and 5 d-glucose (pH 7.4). The aortic rings were first pre-incubated in a normal KRB for 30 min; then the normal KRB was replaced with KRB calcium-free for 5 min before PE (10^−6^ M) was added. Five min after contraction with PE (10^−6^ M), cumulative concentrations of CaCl_2_ (0.1 to 1.0 × 10^−3^ M) were added to the medium. In other experiments, the contraction was induced by 5 × 10^−3^ M BaCl_2_ or 5 × 10^−3^ M TEA for 10 min. BaCl_2_ and TEA are used because they increases vasoconstriction by blocking of potassium channels, thus depolarizing the plasma membrane. In other protocols, the contractile response was induced by an agonist of voltage-dependent calcium channels (10^−8^ M Bay K6844). The aortic rings were pre-incubated with 8-oxo-9-dihydromakomakine (10^−5^ M) for 20 min before the experiment.

### 4.11. Effect Accumulative KCl and Phenylephrine Modulated by 8-Oxo-9-Dihydromakomakine

In the first curve, the aortic rings were stimulated with accumulative KCl doses (10 mM to 60 mM) or PE (10^−9^ to 10^−5^ M). In the second curve, the aortic rings were pre-incubated with 8-oxo-9-dihydromakomakine (10^−5^ M or 10^−4^ M) for 20 min and then stimulated with accumulative KCl doses (10 mM to 60 mM) or PE (10^−9^ to 10^−5^ M).

### 4.12. Statistical Analysis

Data shown in figures and tables are expressed as average ± standard errors of the mean. Statistical analysis was performed by means of one-way and two-way analysis of variance (ANOVA) followed by Bonferroni’s post-hoc test, and some cases Student’s *t*-test. Results are given in the text as probability values, with *p* < 0.05 adopted as the criterion of significance. The graphics and linear regression were done by the least square method, using GraphPad Prism software, version 6.0 (GraphPad Software, Inc, La Jolla, CA, USA).

## 5. Conclusions

This study demonstrates that the natural alkaloid 8-oxo-9-dihydromakomakine obtained from leaves of *Aristotelia chilensis*, a medicinal tree widely employed in Chilean traditional medicine, is able to decrease the tone of arterial smooth muscle. The vasodilator effect of this alkaloid involves responses independent of endothelium, probably due to calcium channels blockage and/or activation of potassium channels, whose mechanism of action remains to be clarified. Our data suggest that Chilean medicinal plants constitute an important reservoir of bioactive compounds that deserves intensive scientific exploration.

## Supplementary Materials

The following are available online, Figure S1. Numbered structure of 8-oxo-9-dihydromakomakine. Figure S2: 1H-NMR (600 MHz, CD_3_OD) spectra of 8-oxo-9-dihydromakomakine. Figure S3. 13C-NMR (150 MHz, CD_3_OD) spectra of 8-oxo-9-dihydromakomakine. Figure S4. gs-H,H-COSY (CD_3_OD) spectra of 8-oxo-9-dihydromakomakine. Figure S5. edited HSQC (CD_3_OD) spectra of 8-oxo-9-dihydromakomakine. Figure S6. gs-HMBC (CD_3_OD) spectra of 8-oxo-9-dihydromakomakine.
