# Supplementary material for: 8-Oxo-9-Dihydromakomakine Isolated from Aristotelia chilensis Induces Vasodilation in Rat Aorta: Role of the Extracellular Calcium Influx
Source: Molecules. 2018 Nov 21;23(11):3050. doi: 10.3390/molecules23113050 (PMC6278248; doi:10.3390/molecules23113050)
Supplement: Supplementary file 1 [file molecules-23-03050-s001.pdf]

molecules

MDPI

Supplementary Materials

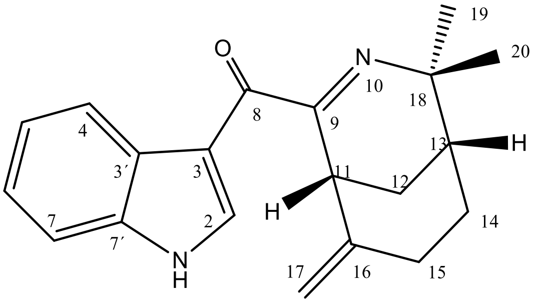

Figure S1. Numbered structure of 8-oxo-9-dihydromakomakine

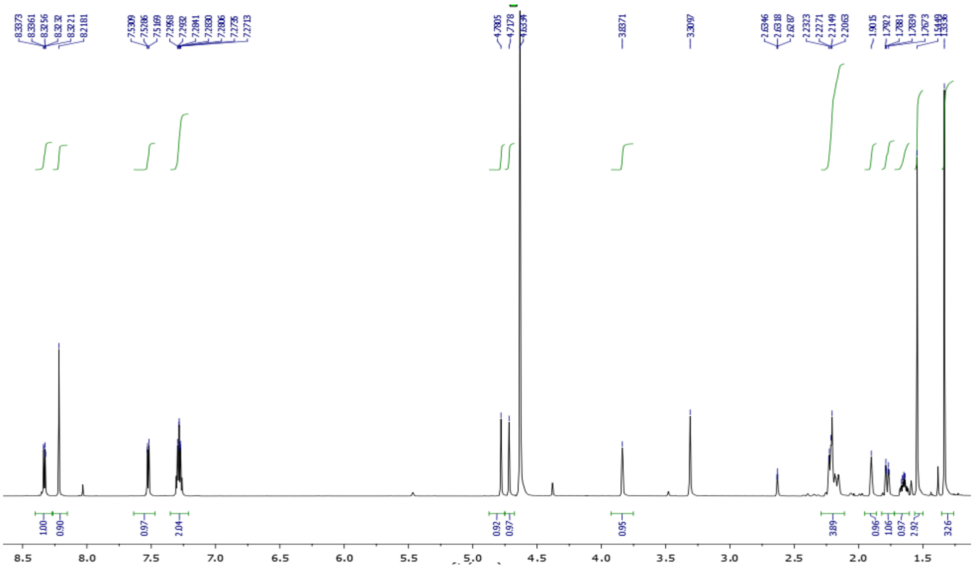

Figure S2. ^{1}H-NMR (600 MHz, CD_3OD) spectra of 8-oxo-9-dihydromakomakine

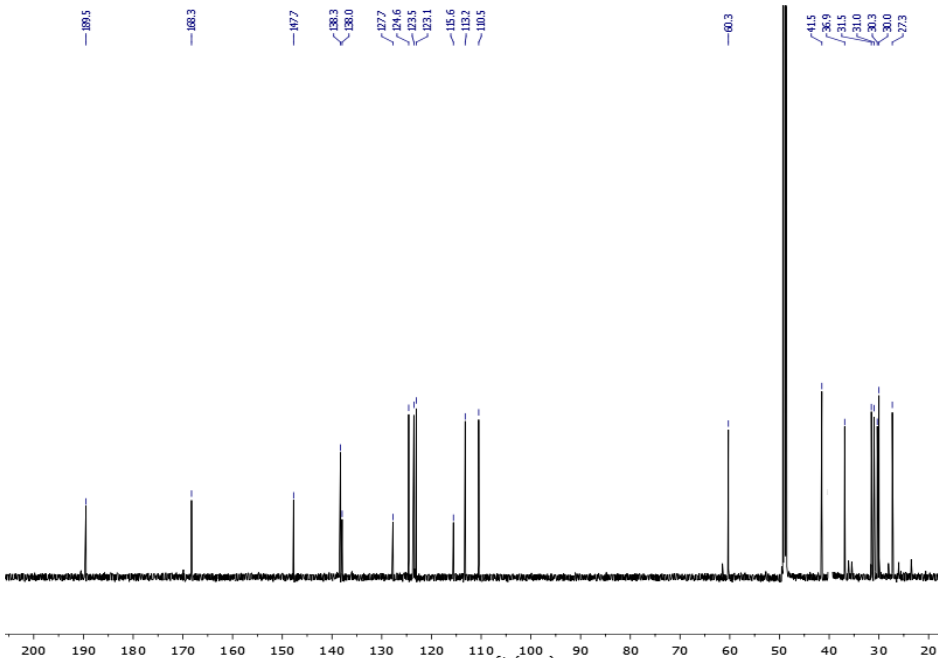

Figure S3. ^{13}C-NMR (150 MHz, CD_3OD) spectra of 8-oxo-9-dihydromakomakine

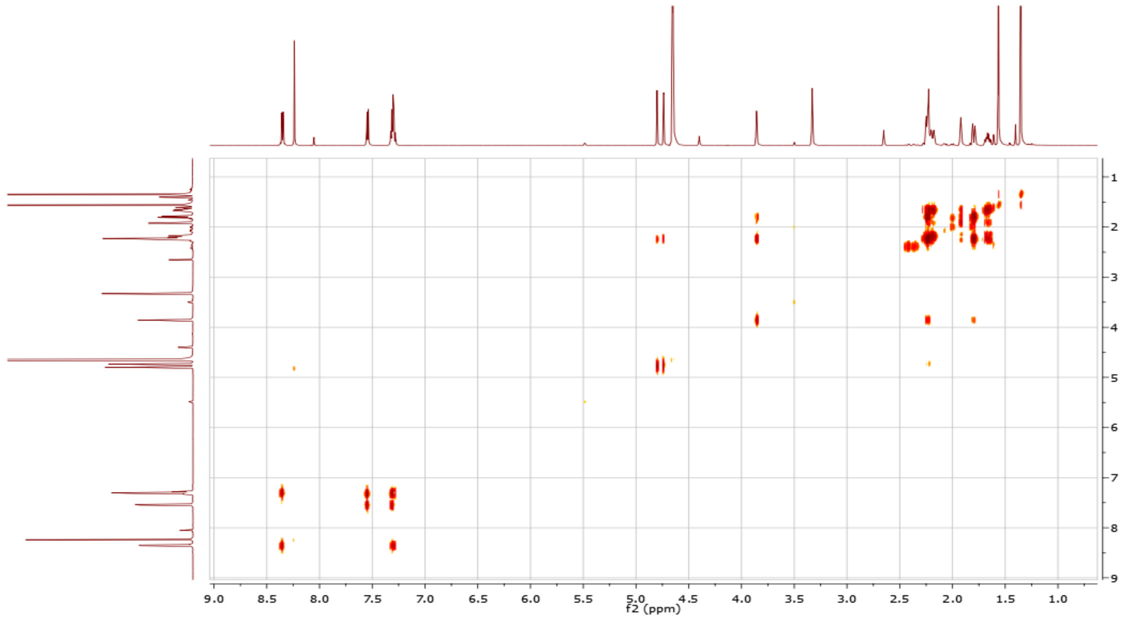

Figure S4. gs-H,H-COSY spectra of 8-oxo-9-dihydromakomakine in CD_3OD

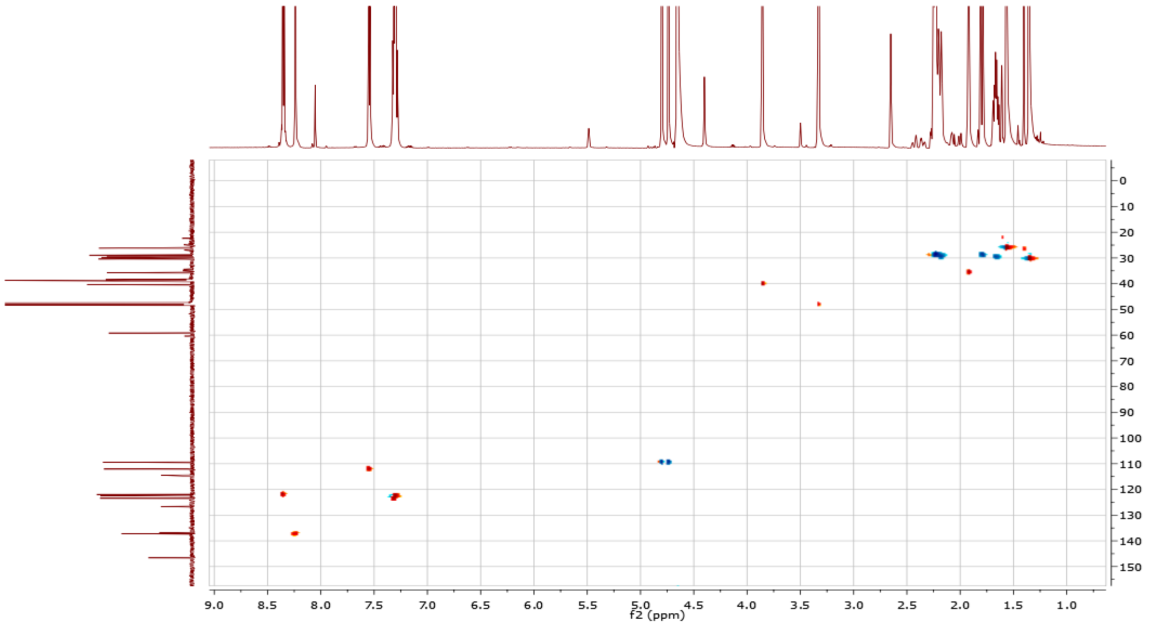

Figure S5. edited HSQC spectra of 8-oxo-9-dihydromakomakine in CD_3OD

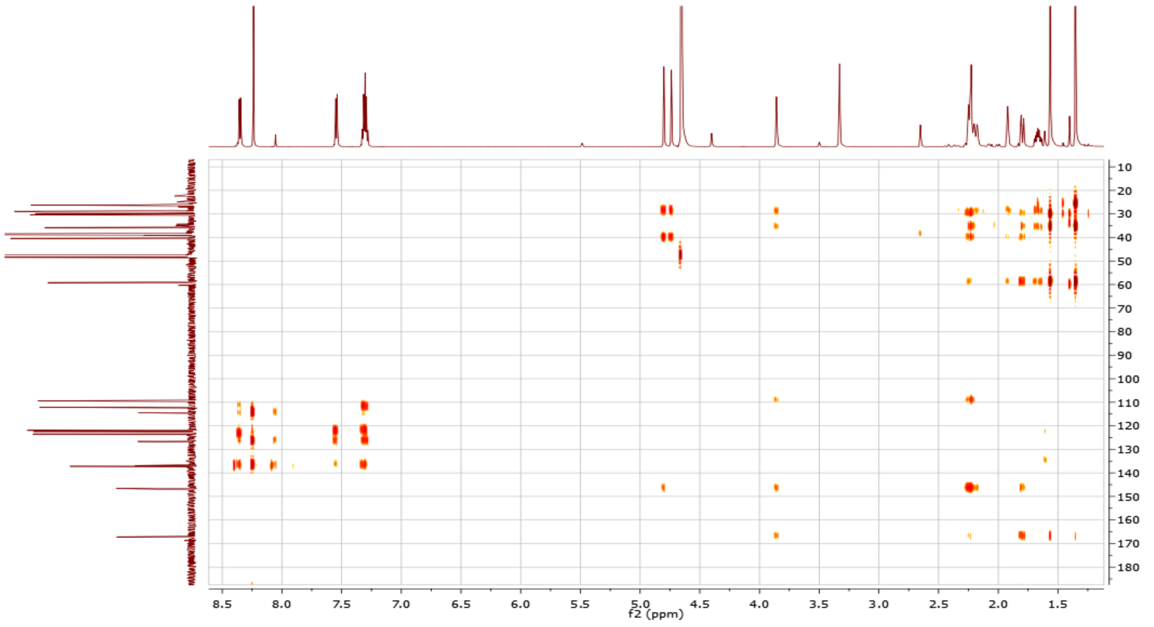

Figure S6. gs-HMBC spectra of 8-oxo-9-dihydromakomakine in CD_3OD
